# The prediction of treatment outcomes by early maladaptive schemas and schema modes in obsessive-compulsive disorder

**DOI:** 10.1186/s12888-014-0362-0

**Published:** 2014-12-25

**Authors:** Nicola Thiel, Brunna Tuschen-Caffier, Nirmal Herbst, Anne Katrin Külz, Christoph Nissen, Elisabeth Hertenstein, Ellen Gross, Ulrich Voderholzer

**Affiliations:** Department of Psychiatry and Psychotherapy, University Medical Center Freiburg, Hauptstrasse 5, 79104 Freiburg, Germany; Department of Clinical Psychology and Psychotherapy, University of Freiburg, Engelbergerstrasse 41, 79106 Freiburg, Germany; Schoen Clinic Roseneck, Am Roseneck 6, 83209 Prien am Chiemsee, Germany

## Abstract

**Background:**

Higher levels of early maladaptive schemas (EMS) and schema modes according to schematherapy by Jeffrey Young are present in obsessive-compulsive disorder (OCD) compared to healthy controls. This study examines the relationship of EMS and schema modes to OC symptom severity and the predictive value of EMS and schema modes on treatment outcome in inpatients receiving Cognitive-Behavioral Therapy (CBT) with Exposure and Response Prevention (ERP). The main assumption was a negative association between the EMS of the domain ‘disconnection’ and dysfunctional coping and parent schema modes and the treatment outcome.

**Methods:**

EMS, schema modes, depression and traumatic childhood experiences were measured in 70 patients with OCD. To analyze the predictors, two regression analyses were conducted considering multiple variables, such as depression, as covariates.

**Results:**

Regression analyses demonstrated that higher scores on the EMS named failure and emotional inhibition and depressive symptom severity at pretreatment were significantly related to poor outcome and explained a high percentage of the variance in OC symptoms at posttreatment. No influence on the treatment outcome was observed for schema modes, other EMS or other covariates.

**Conclusions:**

The results support the approach to extend the CBT with ERP treatment with therapeutic elements focusing on maladaptive schemas, particularly in non-responders.

## Background

Obsessive-compulsive disorder (OCD) is among the most common mental disorders, showing a lifetime prevalence of 2-3% [1]. It is characterized by intrusive thoughts, images or impulses (obsessions) and ritualized repetitive behaviors (compulsions) that cause significant dysfunction and distress. If no adequate treatment is administered, OCD typically takes a chronic course [2]. Cognitive-Behavioral Therapy (CBT) with Exposure and Response Prevention (ERP) is the first-line treatment for OCD according to standard guidelines [2-7]. Meta-analyses confirm that the main components of CBT, cognitive and behavioral interventions such as ERP are comparable in their efficacy [8-11]. More specifically, in meta-analyses, CBT with ERP displays large between group effect sizes of d = 1.3 to 1.5 in comparison with control groups and cognitive restructuring reveals effect sizes of d = 1.1 to 1.5 [2,10-12]. However, it should be taken into account that a strict separation of cognitive interventions in ERP treatment is difficult because cognitive techniques are associated with ERP in some way in most studies [2].

Over the past years, cognitive interventions that have been effective in the treatment of depression and anxiety disorders were adapted to a CBT model addressing typical dysfunctional assumptions in OCD, such as inflated responsibility, perfectionism, overestimation of threat and intolerance of uncertainty [13-18]. However, only a few studies have addressed the origin of these beliefs. Aaron Beck [19] assumed that negative and stressful experiences during childhood may lead to the consolidation of maladaptive core beliefs in individuals, so-called cognitive schemas, which determine affect and behavior. These schemas are assumed to be stable patterns of dysfunctional cognitive processing that may become reactivated by stressful situations [20]. To our knowledge, in OCD, there is only one cognitive treatment approach focusing on schemas, demonstrating a long lasting constellation of inveterate dysfunctional core beliefs in treatment-resistant OCD patients [21]. This integrative schema treatment approach showed clinically significant improvement for 32 patients who were resistant to standard CBT and revealed that maladaptive schemas improved for responders and did not change for non-responders [21].

According to the schema therapy developed by Young [22], schemas named early maladaptive schemas (EMS) are defined as self-perpetuating dysfunctional cognitive patterns that emerge from unmet basic needs and traumatic experiences during childhood [23]. EMS consist of memories, emotions, cognitions and physical sensations that influence thinking and behavior in a dysfunctional way and are stable over time, even after evidence-based treatment of depressed patients [23,24]. Young assumes 18 EMS and groups them into five domains: Disconnection, Impaired Autonomy, Impaired Limits, Other-Directedness and Overvigilance & Inhibition. For a detailed description of the 18 EMS and the domains, please refer to Young and colleagues [23].

Despite the effectiveness of CBT with ERP, studies demonstrate that 17 - 33% of OCD patients do not sufficiently respond to ERP, and 5-29% drop out or refuse treatment [17,25-28]. An increased knowledge about underlying EMS among patients with OCD, particularly among non-responders, is important to gain a deeper understanding of the relationship between core beliefs and treatment outcome and could indicate how to improve the treatment. Some literature has demonstrated the role of EMS in depression, anxiety and eating disorders [24,29,30]. Specifically, increased values in the first EMS domain, *Disconnection*, representing ones expectation that the basic need for security, safety and empathy by others will not be met, are often associated with particularly strong symptomatic impairment, and studies demonstrate a relation to depressive symptom severity [23,24].

Studies relating EMS to OCD are relatively sparse. Three studies examined EMS at a descriptive level [31-33]. In the first study, significantly higher scores in five of 15 EMS were found in OCD in comparison with trichotillomania (so-called *mistrust*, *social isolation*, *shame*, *subjugation* and *emotional inhibition*) [32]. Atalay et al. [31] demonstrated that the EMS questionnaire total score, as well as the schemas social isolation, vulnerability and pessimism, were significantly increased in OCD in relation to healthy controls. Voderholzer *et al.* [33] examined 18 EMS in OCD compared to eating disorder, chronic pain disorder and healthy controls. The patient group could be significantly differentiated from the healthy controls in 17 of the EMS. In addition, OCD patients scored higher on four EMS (*abandonment*, *dependence*, *vulnerability* and *insufficient self-control*) than the eating and chronic pain disorders. In summary, higher levels of EMS in clinical samples compared to healthy controls were proven in all studies. Thus, there is preliminary evidence about the schema construct that inspired us to investigate the predictive value of EMS in treatment outcome. Currently available data are insufficiently stringent to make an accurate prediction. Only one study investigated EMS predictors in 88 OCD patients completing ERP treatment [34]. The EMS named *abandonment* was identified as a negative predictor and the EMS *self-sacrific*e was related to a positive treatment outcome. This interesting study had the limitation that only pretreatment OCD severity and depression were considered as moderating factors in the regression analysis. Other proven predictors in OCD such as hoarding, number of comorbid Axis I disorders, age at onset or gender, which are described as predictors for treatment response in the OCD literature, were not included [56-60]. Moreover, the importance of traumatic life events for the development of OCD is discussed increasingly in the literature [35-40]. Even though findings on the relationship between traumatisation and OCD are still humble, there are results showing higher levels of ‘minor traumatisations’ such as emotional and physical neglect or emotional abuse in OCD [41] Since these traumas have an important part in the development of EMS, the predictive value of traumatisation should be included in the statistical analyses. At this stage, results on the predictive value of traumatisation in OCD are heterogeneous [39,42]. Because these predictors are relevant, our study is of great importance in the replicability of the results of Haaland *et al.* [34] and the extension of the study design.

Since different EMS can be activated at the same time, and because the same patient can show distinct behaviors in specific situations, Young developed so-called schema modes [22]. Schema modes are assumed to be predominant emotional states and coping responses that occur when EMS are triggered. They are assumed to consist of the current emotional and behavioral state of an individual, which can change rapidly and can be functional or dysfunctional [35]. To date, approximately 22 schema modes have been identified and were grouped into four categories: so-called Child modes, Dysfunctional Coping modes, Dysfunctional Parent modes and the Healthy Adult mode [23,44]. Currently, the schema mode concept in Axis I disorders is only rarely examined. Since schema modes can be active during psychotherapeutic sessions, influence the session structure in a negative or positive way and many clinicians align their schematherapeutic work more and more on schema modes than on EMS, it was of great interest to include schema modes in the analysis of this study [43]. Only Voderholzer *et al.* [33] examined these coping responses called schema modes for the first time in OCD and proved that OCD patients scored significantly higher than healthy controls in 10 out of 13 schema modes and higher than the eating and chronic pain disorder group in four schema modes (so-called *vulnerable child*, *angry child*, *punishing parent* and *demanding parent*).

Problematic axis I behaviour such as pathological drinking or gambling, binge eating or obsessive-compulsive behaviour is assigned to the Dysfunctional Coping modes according to the schema theory. They are defined by an overuse of unhealthy coping styles or defense mechanisms, such as avoidance or overcompensation to be distracted by negative emotions [43]. Increased distinct psychopathology and being strongly caught up in the problem behaviour, as it is often the case in OCD patients, implies that the corresponding schema mode is stronger pronounced. Since obsessive-compulsive symptom severity is a negative predictor for treatment outcome and patients with increased levels of these modes, typically show avoiding behaviour such as forgetting sessions or homework, talking about superficialities or discontinue the therapy, we assume that pronounced Dysfunctional Coping modes predict treatment failure. Being in a Dysfunctional Parent mode, patients put extremely high pressure upon themselves or experience self-devaluation and self-hatred. Unhealthy behaviours and destructive rules determine the behavior, something that can be observed in OCD patients as well. Patients in these conditions are often difficult to reach, address problems that they do not work out and frequently reject cooperation in treatment leading to early dropouts in therapy [43]. Based on this definition of Dysfunctional Parent modes, we expect a negative correlation with treatment outcome. In general, it was of great interest to examine, for the first time, the predictive value of schema modes in OCD.

In the present study, we sought to further examine the relationship between EMS and schema modes with OC symptom severity at baseline as well as the predictive value of EMS and schema modes on the treatment outcome in inpatients with OCD receiving CBT with ERP. Three hypotheses will be examined. First, we assume that the degree of EMS and schema modes show a positive relationship with the OCD symptom severity at baseline. Second, we hypothesized that treatment non-responders present higher levels of EMS and schema modes at baseline than responders, and third, we expected that the EMS of the first domain, related to basic safety and high levels of the Dysfunctional Coping and Parent modes, are negatively related to the treatment outcome.

## Methods

### Participants

Eighty-four inpatients diagnosed with OCD were recruited from the Department of Psychiatry and Psychotherapy, University Medical Center in Freiburg and the Schoen Clinic Roseneck in Prien. The inclusion criteria were ages between 18 and 65 years and a primary diagnosis of OCD as assessed by the Structured Clinical Interview for DSM-IV (SCID-I) [45]. The SCID-I was administered by trained and experienced raters. All raters attended a SCID-I and –II training consisting of a two-day theoretical training and scoring videos by a certified trainer for SCID. The inpatients were excluded if they had a primary diagnosis other than OCD, a current or lifetime history of psychotic episodes, substantial neurological impairment, severe cognitive dysfunction, acute suicidal symptoms and insufficient German language skills. Four patients refused to participate in the investigation, four were excluded due to other primary diagnoses, two due to cognitive dysfunction, two because of comorbid psychotic episodes and two could not fill out the questionnaires because of compulsive behavior. Thus, 70 inpatients fulfilled the inclusion criteria and were included in the present study. Three patients dropped out during treatment at their own request and did not participate in the posttreatment evaluation. A detailed description of the demographic and clinical characteristics is presented in Table 1.

**Table 1:** **Demographic and clinical characteristics of the sample (N = 70)**

**Demographics**	**M (SD)**
Age in years	35.3 (11.1)
Years of education	15.5 (3.2)
	**N (%)**
Females	43 (61.4)
Marital status	
Single	53 (76)
Married or partnered	13 (19)
Divorced or separated	4 (6)
Employment status	
Employed	35 (50)
Studying	15 (21)
Disability pension	9 (12)
Unemployed	8 (11)
**Clinicals**	**N (%)**
At least one comorbid Axis I diagnosis	53 (75.7)
Major depressive disorder	29 (41.2)
Agoraphobic or panic disoder	11 (15.7)
Social- or specific phobia	6 (8.6)
Generalized anxiety disorder	3 (4.3)
PTSD	3 (4.3)
Eating disorder	4 (5.7)
Somatoform disorder	1 (1.4)
	**M (SD)**
Age of onset	19.7 (9.9)
OCI-r^a^	29.6 (12.6)
BDI^b^	20.3 (12.7)
CTQ^c^	47.2 (18.4)
Y-BOCS^d^ post-treatment	14.55 (7.02)

Note: ^a^OCI-r: Obsessive Compulsive Inventory-revised, ^b^BDI: Beck Depression Inventory, ^c^CTQ: Childhood Trauma Questionnaire, ^d^Y-BOCS: Yale-Brown Obsessive-Compulsive Scale.

At pretreatment, the sample was characterized by moderate to severe levels of obsessive-compulsive symptom severity (*M* = 23.97, *SD* = 5.28) according to the Yale-Brown Obsessive-Compulsive Scale (Y-BOCS) [46]. The mean duration of the OCD in years was 15.1 (*SD* = 11.3). The study was approved by the local Ethics Committee for research with human subjects. Written informed consent was obtained from all participants prior to baseline assessment after the rationale of the study was fully explained to the subjects.

### Measures

#### Y-BOCS

The Y-BOCS [46] is a semi-structured, clinically-administered interview that is considered the gold standard to assess OCD symptom severity [47,48]. It has demonstrated a high inter-rater reliability, internal consistency and convergent validity [46,49]. The first 10 items were used as primary outcomes and are suitable to demonstrate symptom changes over the course of treatment [50]. Internal consistency for baseline scores for the current sample was α = 0.89.

#### Young Schema Questionnaire - Short Form 3 (YSQ-S3)

The YSQ-S3 [51] is a 90-item self-report instrument that investigates the presence of 18 EMS with five items per scale. Each item is a statement of a character issue that the patient scores on a 6-step Likert-type response format ranging from *completely untrue for me* to *describes me perfectly*. Higher scores indicate a stronger presence of the respective schema. Adequate reliability, convergent, factorial and discriminant validity have been demonstrated for the German version [52]. In the current sample, Cronbach’s alpha for the baseline YSQ-total was α = 0.96.

#### Schema Mode Inventory (SMI-r)

The SMI-r [53] is a 124-item self-report questionnaire that investigates the presence of 14 schema modes with 4 to 10 items in each subscale. Each item is rated on a 6-step Likert-type scale. Higher scores indicate a stronger presence of the schema mode. The so-called *perfectionistic* and the *suspicious overcontroller* mode were added as explorative scales because the manifestation of the *perfectionistic overcontroller* mode seemed particularly interesting in patients with OCD. For a brief description of the schema modes, see Young, Klosko and Weishaar [23]. The German version demonstrated good-to-excellent internal consistency and construct validity. Furthermore, the 14-factor structure was approved [54]. Internal consistency for baseline scores for the current sample was α = 0.91.

#### Beck Depression Inventory-II (BDI-II)

The BDI-II [55] is a well-known 21 item self-report measure of the severity of depressive symptoms. It has demonstrated high internal consistency, test-retest reliability and construct validity that also applies to the German version [56]. In the current sample, Cronbach’s alpha for the BDI at baseline was α = 0.93.

#### Obsessive Compulsive Inventory-revised (OCI-r)

The OCI-R [57] is a self-report measure for assessing symptoms of OCD. It contains 18 Items and six subscales and has good psychometric properties [57-59]. These apply likewise for the German version [60]. In the current sample, Cronbach’s alpha for the OCI-r total score at baseline was α = 0.82.

#### Childhood Trauma Questionnaire (CTQ)

The CTQ [61] is a 31-item self-report instrument that retrospectively assesses the subjective frequency of five forms of childhood trauma experienced with good psychometric properties. The CTQ measures five domains: emotional abuse, physical abuse, sexual abuse, emotional neglect and physical neglect. For the German version, the factor structure, good reliability and validity were demonstrated [62]. In the current sample, Cronbach’s alpha for the CTQ total score at baseline was α = 0.67.

#### Life satisfaction

Life satisfaction was assessed by a self-rating ranging from ‘very dissatisfied’ (1) to ‘very satisfied’ with my life’ (10).

### Procedure

Every patient admitted to one of the two hospitals for an OCD treatment was informed about the study. If the patient agreed to participate, the Axis I disorders diagnoses were confirmed by the SCID-I within the first seven days of treatment. If the inpatient met the inclusion criteria, the severity of the OC symptoms was assessed based on the Y-BOCS, and the questionnaires (see 2.2) were handed out. All patients participated in a multimodal inpatient CBT with ERP treatment for OCD according to the treatment manual by Lakatos and Reinecker [63]. The treatment consisted of twice-weekly individual therapy sessions (50 min each) and a weekly educational group (90 min) conducted by experienced therapists having weekly team meetings to discuss ongoing cases and difficulties. After the general education regarding OCD and the development of an individual disease model, a fear hierarchy was constructed. The exposure began with moderately anxiety-provoking situations and increased to the most distressing fear and was conducted therapist-accompanied within the therapy sessions as well as in homework assignments. The ERP was combined with the identification of negative beliefs and appraisals as well as cognitive restructuring. A total of 46 inpatients (65,7%) received selective serotonin-reuptake inhibitors (SSRI) or selective serotonin-noradrenalin reuptake inhibitors (SNRI). The mean duration of the inpatient stay in the current sample was 10.6 weeks (*SD* = 4.4), 21 individual therapy sessions. OC symptom severity was again evaluated posttreatment.

### Data analysis

To test the first hypothesis, correlation analyses were conducted to investigate the relationship between EMS, schema modes and obsessive-compulsive symptoms at pretreatment. To verify the second hypothesis, in a first step, responders were defined as those subjects who showed clinically significant change (CSC) after treatment according to the two-fold criterion provided by Jacobson and Truax [64]. CSC is fulfilled if (a) a symptom score is under a calculated cut-off score at posttreatment and (b) a symptom score had decreased by a reliable amount of change exceeding the measurement error (reliable change index (RCI)). The RCI was calculated based on the test-retest reliability of the Y-BOCS (r = 0.61) according to Woody *et al.* [49]. The calculated cut-off score in the present study to determine the non-clinical range was Y-BOCS = 13 or below [64]. To achieve CSC and therefore be classified as a responder, a patient’s individual change score had to be above 1.96 and the post Y-BOCS score had to be 13 or less. In a second step, an exploratory one factorial multivariate analysis of variance (MANOVA) was computed investigating the distribution of the EMS, schema modes and other variables in the responder- and non-responder groups. The third hypothesis concerning the identification of EMS and schema modes as predictors of treatment outcome was tested based on two regression analyses. Stepwise multivariate regression analyses were conducted to test whether EMS and schema modes predicted treatment outcome using the posttreatment Y-BOCS as the dependent variable. Because increased depression rates affect the completion of self-rating questionnaires, depressive symptom severity (BDI-II) was included as a covariate. Furthermore, in all statistical calculations, we controlled for several covariates. Since baseline obsessive-compulsive symptom severity (Y-BOCS), number of comorbid Axis I disorders (SCID-I), age at onset (first onset of symptom measured by self-rating and alignment with data from previous clinical reports), gender and hoarding subtype (OCI-r) are consistent described as negative predictors for treatment response in the OCD literature, they were taken into account [65-69].

Traumatisation (CTQ) is considered due to the possible relevance of the development of EMS. Although specific personality disorders (Cluster A, schizotypal, narcissistic, two or more comorbid personality disorders) are associated with poor treatment outcome in patients with OCD, these predictors were not included in the analyses because too few patients presented these specific personality disorders [70].

The statistical assumptions for the regression analyses were verified with residual plots and histograms for residuals, which showed a normal distribution of the residuals. Prior to the regression analyses, multicollinearity among the predictor variables was statistically investigated by computing Variance inflation factors (VIF). As a general rule, a VIF above the cut-off value of 10 indicates a collinearity problem [71]. The VIF was above 1.8 in none of the predictor indicating no significant multicollinearity problem. In all analyses, the level of significance was set at p ≤ 0.01 (two-tailed tests). The Statistical Package for Social Sciences (SPSS), version 18, was used for all calculations. Inter-rater reliability for the Y-BOCS was determined for a subset of 5 patients with two raters from Freiburg sitting in the same room, with no communication between the two during the interview. The inter-rater reliability between the two raters was high with intraclass correlation coefficients (ICCs) of >0.86.

## Results

### Hypothesis 1: Positive correlations of EMS and schema modes with the OC symptom severity at baseline

As expected, significant positive correlations of the EMS and schema modes with the Y-BOCS prior to treatment (Total score: YSQ *ρ* = 0.26; *p* = 0.014 and SMI r = 0.25, *p* = 0.018), as well as a highly positive correlation with the OCI-r pre (Total score: YSQ *ρ* = 0.45; *p* < 0.001 and SMI *r* = 0.44, *p* < 0.001), were proven.

### Hypothesis 2: Higher levels of EMS and schema modes in the non-responder than in the responder group

Analyses with paired sample t-tests showed a significant reduction of obsessive-compulsive symptoms (T = 10,006; df = 67; *p* < 0.001) in the Y-BOCS from pre- (*M* = 24, *sd* = 6.2) to posttreatment (*M* = 14.6, *sd* = 7.0). The mean Y-BOCS reduction was 37,5%, indicating a positive outcome of the ERP and pharmacotherapy treatment, as a symptom reduction of 35% in the literature is designated as treatment response [3]. According to the 35% symptom reduction criterion, 53% of the patients (N = 37) achieved treatment response, and 43% (N = 30) were non-responders. According to the criteria of clinically significant change (CSC), response was achieved in 27 of the 67 patients who completed the treatment (38,6% responders; 57,1% non-responders).

In an exploratory one factorial MANOVA, the distributions of the EMS, schema modes and other variables in the responder- and non-responder groups were investigated (see Table 2). The MANOVA yielded a significant main effect in the YBOCS post between responders and non-responders (F = 76.6; *p* < 0.001) and not in the YBOCS pre. Moreover, non-responders had significantly higher pretreatment scores on four EMS (*emotional inhibition*, *social isolation*, *mistrust/ abuse* and *defectiveness*) and 4 schema mode variables (*vulnerable child*, *detached protector*, *bully and attack* and schema mode global score). In the schema mode named *happy child*, non-responders showed significantly lower scores at pretreatment than responders. For all other EMS and schema modes, the differences were not significant. Concerning psychopathological scores, non-responders showed a significantly lower score in depression symptom severity and lower scores in life satisfaction at posttreatment. For complete results, see Table 2.

**Table 2:** **Comparison of psychopathology scores, early maladaptive schemas and schema modes between responders and non-responders**

	**M ± SD responders**	**M ± SD non-responders**	**F-value**	**p-value**
Y-BOCS^a^ (pre)	24.6 ± 6.2	23.6 ± 6.3	0.44	.509
OCI-R^b^ (pre)	30.1 ± 12.6	30.1 ± 12.6	0.00	.983
BDI^c^ (pre)	16.5 ± 10.7	23.5 ± 13.3	5.18	.026*
CTQ^d^ (pre)	43.0 ± 15.5	50.4 ± 20.0	2.63	.110
life satisfaction (pre)	4.2 ± 1.9	3.9 ± 2.2	0.33	.568
life satisfaction (post)	7.0 ± 1.7	4.9 ± 1.8	23.86	<.001***
age of onset	22.1 ± 9.3	18.5 ± 10.2	2.09	.154
treatment duration	14.0 ± 10.8	11.1 ± 3.2	2.57	.114
**EMS (Domain, YSQ** ^**e**^ **scales)**				
D^g^: Emotional deprivation	2.5 ± 1.4	2.7 ± 1.4	0.23	.637
D^g^: Abandonment	4.0 ± 1.2	3.7 ± 1.2	0.85	.359
D^g^: Mistrust/abuse	2.5 ± 0.9	3.2 ± 1.2	6.75	.012*
D^g^: Social isolation	2.7 ± 1.3	3.7 ± 1.6	7.44	.008**
D^g^: Defectiveness	2.5 ± 1.2	3.2 ± 1.4	4.23	.044*
IA^h^: Failure	2.7 ± 1.2	3.3 ± 1.4	3.53	.065
IA^h^: Dependence	3.0 ± 1.0	3.0 ± 1.2	0.00	.986
IA^h^: Vulnerability	2.6 ± 1.1	3.0 ± 1.0	1.94	.169
IA^h^: Enmeshment	3.1 ± 1.4	2.8 ± 1.1	0.65	.424
IL^i^: Entitlement	2.5 ± 0.7	2.8 ± 1.0	1.17	.284
IL^i^: Insufficient self-control	3.1 ± 0.9	3.4 ± 0.9	2.723	.104
OD^j^: Subjugation	3.3 ± 1.2	3.6 ± 1.6	1.45	.232
OD^j^: Self-sacrifice	3.5 ± 1.2	3.4 ± 1.0	0.05	.826
OD^j^: Approval-seeking	3.5 ± 1.0	3.6 ± 1.2	0.22	.643
OI^k^: Emotional inhibition	2.5 ± 0.8	3.2 ± 1.1	8.35	.005**
OI^k^: Unrelenting standards	4.0 ± 1.0	4.2 ± 0.9	0.84	.364
OI^k^: Negativity	3.1 ± 1.4	3.5 ± 1.0	2.23	.141
OI^k^: Punitiveness	3.3 ± 1.2	3.6 ± 1.0	0.93	.337
YSQ^e^-total score	3.0 ± 0.7	3.3 ± 0.8	2.94	.091
**Schema modes (SMIr** ^**f**^ **)**				
CM^l^: Vulnerable child	2.7 ± 0.9	3.4 ± 1.1	8.63	.005**
CM^l^: Angry child	2.6 ± 0.7	3.0 ± 0.9	3.08	.084
CM^l^: Enraged child	1.4 ± 0.5	1.4 ± 0.5	0.09	.765
CM^l^: Impulsive child	2.2 ± 0.6	2.3 ± 0.8	0.66	.420
CM^l^: Undisciplined child	2.6 ± 0.6	2.7 ± 0.6	1.25	.268
CM^l^: Happy child	3.4 ± 0.9	2.9 ± 0.8	5.05	.028*
DCM^m^: Avoidant protector	3.4 ± 0.8	3.4 ± 0.8	0.08	.775
DCM^m^: Detached protector	2.1 ± 0.7	2.6 ± 1.0	7.29	.009**
DCM^m^: Self-soother	3.0 ± 0.9	3.3 ± 1.0	1.61	.210
DCM^m^: Self-aggrandizer	2.4 ± 0.7	2.6 ± 0.7	0.80	.373
DCM^m^: Bully and attack	1.6 ± 0.5	1.9 ± 0.7	4.19	.045*
DCM^m^: Perf. overcompensator	3.6 ± 0.8	3.9 ± 0.7	1.61	.209
DCM^m^: Susp. overcontroller	2.6 ± 0.7	2.9 ± 0.8	3.26	.075
DPM^n^: Punishing parent	2.2 ± 1.0	2.5 ± 0.9	1.27	.263
DPM^n^: Demanding parent	4.1 ± 0.9	4.1 ± 0.9	0.01	.940
Healthy adult	3.7 ± 0.9	3.7 ± 0.7	0.12	.730
SMIr^f^-total score	2.6 ± 0.4	2.9 ± 0.5	6.06	.016*

Note: ^a^Y-BOCS: Yale-Brown Obsessive-Compulsive Scale, ^b^OCI-r: Obsessive Compulsive Inventory-revised, ^c^BDI: Beck Depression Inventory, ^d^CTQ: Childhood Trauma Questionnaire, ^e^YSQ: Young Schema Questionnaire, ^f^SMIr: Schema Mode Inventroy-revised, ^g^D: 1. Domain Disconnection, ^h^IA: 2. Domain Impaired Autonomy, ^I^IL: 3. Domain Impaired Limits, ^j^OD: 4. Domain Other-Directedness, ^k^OI: 5. Domain Overvigilance & Inhibition, ^l^CM: Child Mode, ^m^DCM: Dysfunctional Coping Modes, ^n^DPM: Dysfunctional Parent Mode, *p < .05, **p < .01, ***p < .001.

### Hypothesis 3: Predictive value of pretreatment EMS and schema modes on treatment outcome

In a first step, two separate stepwise multivariate regression analyses were computed to reduce the large number of predictors [72]. First, the 18 EMS and second, the 16 schema modes were entered as predictors, while the posttreatment Y-BOCS acted as the dependent variable. Variables with a significance level below p < 0.1 were included in further analyses. Concerning the EMS variables, this was obtained for the EMS constructs of *emotional inhibition*, *failure* and *emotional deprivation*. For the schema modes, the *vulnerable child* and *perfectionistic overcompensator* met these criteria.

In a second step, two stepwise multivariate regression analyses were calculated including the set of covariates described in 2.4. The aim was to identify the independent involvement of each variable in the prediction of the treatment outcome. The first regression explored the impact of the three EMS and the controlling variables. The EMS *failure* explained 21% of the variance of the treatment outcome, while *emotional inhibition* explained an additional 6%. The set of covariates and the EMS *emotional deprivation* did not make a significant contribution.

In the second regression, the predictive value of two schema modes and the covariates on the treatment outcome was computed. Depressive symptom severity was the only variable approved in the analysis, explaining 20% of the variance. The remaining variables were excluded as predictors. A summary of the regression analyses is provided in Table 3.

**Table 3:** **Statistics of the regression analyses with post-treatment Y-BOCS** ^a^ **regressed by multiple variables (N = 67)**

			***F***	***R*** ^**2**^	***R*** ^**2**^ **corr.**	***B***	**SE** ***B***	***β***	***T***
1. Regression analysis	Model 1	Failure	17.31	0.22	0.21***	2.42	0.58	0.47	4.16***
	Model 2	Failure & emotional inhibition	12.35	0.29	0.27***	1.66 1.89	0.64 0.78	0.32 0.31	2.59* 2.44*
		Emotional deprivation						−0.22	−1.56
		Y-BOCS^a^ pre						0.17	1.45
		BDI^b^ pre						0.17	1.17
		CTQ^c^ pre						−0.01	−0.07
		Hoarding						0.15	1.23
		No.CD^d^						0.01	0.11
		Gender						0.15	1.41
		Age at onset						−0.09	−0.84
2. Regression analysis	Model 1	BDI pre	15.74	0.21	0.20***	0.25	0.06	0.46	3.97***
		Y-BOCS^a^ pre						0.17	1.29
		Vulnerable child						0.27	1.83
		Perfectionistic overcompensator						0.18	1.51
		CTQ^c^ pre						0.02	0.18
		Hoarding						0.18	1.44
		No.CD^d^						0.00	0.01
		Gender						0.13	1.12
		Age at onset						−0.15	−1.31

Note: ^a^Y-BOCS: Yale-Brown Obsessive-Compulsive Scale, ^b^BDI: Beck Depression Inventory, ^c^CTQ: Childhood Trauma Questionnaire, ^d^No.CD: Number of comorbid Axis I disorders, *p < .05, **p < .01, ***p < .001.

## Discussion

The present findings on the EMS and schema mode construct in OCD extend previous research. Consistent with our first hypothesis, we demonstrated significant positive correlations between the degree of EMS and schema modes with the severity of obsessive-compulsive symptoms. The second hypothesis received mixed support. Statistically higher levels of EMS in the non-responder group could only be detected in four out of 18 EMS, but responders and non-responders did not differ in the EMS total score. Concerning schema modes, non-responders presented significantly higher scores in 4 out of 15 variables and on the schema mode global score compared to responders. The third hypothesis concerning the predictive value of EMS of the first domain and the Dysfunctional Coping and Parent modes could not be confirmed. Although non-responders showed significantly stronger presence of three out of five EMS in first domain, these EMS could not be identified as treatment predictors. However, findings demonstrated that higher scores on the EMS *failure*, of the second domain, and *emotional inhibition*, of the fifth domain, were related to poorer outcomes in ERP treatment for OCD. Concerning the calculations regarding the schema modes, only depressive symptom severity was identified as a negative predictor.

In the following, the identified predictors will be discussed in detail. Young and colleagues [23] theorized that the EMS *failure* involves the perception that one will inevitably fail or is less successful than others. Moreover, persons who score high on this schema often assume that they are inept or untalented. Understandably persons with these assumptions about themselves interfere with their own efforts in therapy and thus negatively influence the treatment outcome. The expectation of slight success becomes a self-fulfilling prophecy, as discussed in the concept of perceived self-efficacy [73]. A considerable number of studies demonstrated the role of higher treatment expectations in improved outcomes [74-76], whereas low expectations were associated with poor treatment outcomes in CBT of anxiety and depressive disorders [77-79]. Clinicians working with patients presenting a high *failure* EMS should articulate the typical assumptions which are accompanied with this EMS to prevent the reconfirming of the EMS. In OCD treatment, results regarding treatment expectations are inconsistent and refer to a limited number of studies with small sample sizes [74,80-82]. Our results support the assumption that negative expectations about failure affect treatment success adversely. In addition, previous research has shown that *failure* was one of the five EMS explaining most of the variance in anxiety symptoms, and cognitive avoidance was predicted by *failure* in people suffering from posttraumatic stress [83,84]. Furthermore, *failure* predicted depressive symptoms and anxiety in nonclinical samples and was associated with depression severity [85,86].

Individuals scoring high on the so-called *emotional inhibition* schema typically inhibit the expression of feelings to avoid disapproval by others or shame, seem very controlled and aim for perfect self-regulation [23]. Typically, anger, aggression, joy, sexual excitement and play are inhibited. In ERP treatment feared stimuli are confronted, the experience of anxiety is desired and the therapist supports emotional processes [63]. The inhibition of feelings is often considered as an indication of incorrect treatment or a lack of involvement of the patient. In line with these principles of ERP, our results indicate that the suppression of emotions adversely affects the treatment outcome and supports the concept of experiential avoidance. Therapists should be aware, if this EMS is prevalent in the patient, to counteract treatment failures and should take time to build up a strong therapeutic relationship to encourage inhibited patients to reveal emotions and guide them in emotion regulation without the use of maladaptive coping strategies.

Also in the present study, non-responders scored significantly higher on this EMS prior to treatment than responders. Furthermore, the *emotional inhibition* EMS was previously declared as one of the key EMS in obsessive-compulsive, anxiety and avoidant personality disorders, explained a great part of the variance of anxiety symptoms, correlated significantly with PTSD symptoms and was identified as a negative predictor in bipolar disorder [32,84,87-92]. In addition, this EMS appeared to be more resistant to short-term SSRI treatment than other EMS in a sample of major depressive patients [104].

Our results regarding the EMS as predictors for treatment outcomes in OCD are not in line with previous results identifying the *abandonment* and *self-sacrifice* schemas as outcome predictors [34]. This may be because these authors only considered two covariates in their analyses. Moreover, the participants presented only mild depressive symptoms and only a few fulfilled the criteria for a depressive disorder. Furthermore, the results might also diverge due to cultural influences that may exist between Norwegian and German samples.

It should be taken into consideration that the encountered results regarding the EMS may not only due to the obsessive-compulsive psychopathology, but also to the moderate depression severity of the patients since Atalay *et al.* [101] demonstrated that depressive dispositions activated EMS rather than anxious dispositions. Moreover, it was proven that already short-term SSRI treatment reduced EMS activation levels for some EMS significantly especially for patients suffering from severe depressive symptoms [101].

Studies investigating schema modes in Axis I disorders are very limited, with a total of only two studies [33,53,63,64]. The present study revealed no relation between schema modes and treatment outcomes. Schema modes reflect a current state rather than a trait and are less stable than EMS [23]. An explanation for the missing relation to the treatment outcome could be that patients were not triggered by situations while answering the questionnaires and thus the schema modes were inactive. Depression severity emerged as the most prominent predictive factor for treatment failure in the analyses of the schema modes but not in the calculations concerning the EMS. Even prior to treatment, non-responders presented significantly higher depression values than later responders. According to the literature, the predictive value of depressive symptoms is inconclusive with studies observing a relation to treatment failure [65,67,69,95] while others did not [12,81,96,97,99]. However, severe depression and the presence of a Major Depressive Disorder (MDD) are continuously linked with negative treatment outcomes [98,100]. In our study, 41.2% of the patients fulfilled the criteria for a MDD, with the majority of patients (58.6%) suffering from moderate MDD. This explains why some part of the patients received additional pharmacotherapy treatment. Abramowitz [98] assumes that the negative impact of depression on treatment outcome could be explained by the over-excitation some depressive patients present, as this negatively affects the habituation process during exposure therapy. Others assume that patients with OCD and comorbid depression are not sufficiently motivated for the challenging ERP treatment, suffer more severe distress and functional impairment or have a greater disposition to misinterpret innocuous intrusive thoughts as being significant [68,102]. Identifying depressive symptom severity as a negative predictor for treatment outcome is thus in line with one aspect of previous research results. The number of comorbid axis I diagnoses was not associated with treatment outcome in the present study most likely because all axis I diagnoses were taken into account. Our results indicate that depressive symptoms in OCD must receive greater attention and be treated in more detail.

The limitations of this study include the use of merely self-report measures to assess EMS and schema modes. Self-report measures only allow the assessment of conscious aspects of EMS and schema modes. Furthermore, those subjectively measured constructs are surveyed retrospectively. Further possible difficulties with this type of measurement are strategic reporting, response styles and the feeling of shame. Another limitation is the relatively small sample size compared to the number of predictors included in the analysis. Thus, the risk for unstable linear regression models and the possibility for type I errors are enhanced. A replication with larger sample sizes is needed to demonstrate the robustness of the identified EMS predictors. Moreover, no follow-up data were gathered. In the future, the investigations of long-term effects of EMS and schema modes on the treatment outcome in OCD are desirable. Moreover, previously identified outcome predictors in OCD treatment, such as treatment motivation, patient adherence and expectations, low insight and expressed emotions were not included in the study because these variables were not gathered. They may explain some of the variance of the present predictors *failure*, *emotional inhibition* and depression [68]. Besides, the identification of a predictor may lead to unwarranted hypotheses about possible causalities. Lastly, no formal fidelity analyses were conducted.

The strengths of the present study are that comorbidities, such as severe depressive or personality disorders, were not excluded, as in most investigations. As a result, it can be assumed that the examined OCD sample is representative, which leads to the increased generalizability of the results. Furthermore, important variables that were identified as predictors for treatment outcome in previous studies, such as depression, axis I comorbidity, traumatization, etc. were included in the current statistical analysis to identify potential influences. In addition, the results are based on a phase IV study conducting the effective first-line treatment CBT with ERP for OCD under real inpatient treatment conditions treating seriously burdened patients.

## Conclusions

The present study successfully detected negative predictors in OCD treatment. Based on the results, we suggest the application of the EMS questionnaire and BDI-II prior to treatment in OCD patients to identify the degree of the EMS *failure* and *emotional inhibition* together with depression as potential negative influencing treatment variables. Since non-responders additionally showed a higher activation level in three EMS of the first domain, *emotional inhibition* and different schema modes compared to responders, the awareness about the activation levels of EMS and schema modes may provide an indication of patients responding well and poor to treatment. If clinicians are aware of potential non-responders at an early stage of treatment, an adjustment of the treatment is possible to generate a more satisfactory treatment outcome and to minimize subsequent treatments. But there is good news for clinicians working with patients with a general higher EMS activation level. Based on the data, we assume that these patients will not be automatically non-responders.

As described above, CBT with ERP is successful in the treatment of OCD. Changes in clinical symptoms and most likely the underlying schemas are achieved, but non-responders exist and relapses are known. Because schema therapy (ST) was especially developed for patients not responding optimally to traditional CBT, and because first studies yielded good results [23,103,104], the use of schema therapeutic elements in the treatment of OCD could help to specifically treat the identified negative EMS predictors and subsequently further improve the treatment outcome. Particularly in patients presenting the EMS *emotional inhibition*, a schema therapeutic approach might be beneficial, because it is an emotion-focused method that prevents emotional avoidance through the use of techniques named *imagery rescripting* or *chair work*. Future research would benefit from the examination of the application of the schema therapeutic approach in CBT or the comparison of CBT and ST in their efficacy in OCD treatment, especially in non-responders. To date, only one study concerning axis I disorders exists, which compares ST with traditional CBT in veterans suffering from PTSD and demonstrates ST to be more effective [87]. Lastly, further studies should examine to what extent depressive symptom severity is a mediator or moderator of EMS and schema modes based on a mediator analysis with a predefined structural equation model.
